# Infestation Pattern and Population Dynamics of the Tropical Bed Bug, *Cimex hemipterus* (F.) (Hemiptera: Cimicidae) Based on Novel Microsatellites and mtDNA Markers

**DOI:** 10.3390/insects11080472

**Published:** 2020-07-25

**Authors:** Wan Nur Fatanah Wan Mohammad, Li-Shen Soh, Wan Nurainie Wan Ismail, G. Veera Singham

**Affiliations:** 1 Centre for Chemical Biology, Universiti Sains Malaysia, Bayan Lepas 11900, Penang, Malaysia; fatanahwm@gmail.com (W.N.F.W.M.); slshen_93@hotmail.com (L.-S.S.); 2 Faculty of Resource Science and Technology, Universiti Malaysia Sarawak, Kota Samarahan 94300, Sarawak, Malaysia; wiwnurainie@unimas.my

**Keywords:** bed bug, *Cimex hemipterus*, microsatellite, mtDNA, population genetics, infestation dynamics

## Abstract

The tropical bed bug, *Cimex hemipterus* (F.), has now emerged as an important public health pest in the tropics. Despite its alarming infestation rate, the information on its population genetics remains scarce. Here, we described the infestation structure and population dynamics of *C. hemipterus* in the tropics, especially Malaysia and Singapore, based on eight novel microsatellites and two mtDNA markers, including cytochrome c oxidase I (COI) and 16S rRNA genes. Across populations, microsatellite data revealed high genetic diversity with significant genetic differentiation and restricted gene flow. Analysis within populations revealed evidence of a recent bottleneck. Nonetheless, elevated genetic diversity in nearly all populations suggests that the propagule in *C. hemipterus* populations were much diverse, distantly related (mean *r* = 0.373), and not significantly inbred (mean *F*_IS_ = 0.24) than that observed in *Cimex lectularius* from previous studies. We observed seven mtDNA haplotypes across the 18 populations studied (Hd = 0.593) and several populations displayed more than one matrilineal descent. The two markers were generally congruent in suggesting a common, genetically diverse (especially at the nuclear region) source population with possibilities of multiple introductions for the bed bug populations in the present study.

## 1. Introduction

The bed bug is a nocturnal blood-sucking ectoparasite that has now emerged as an important public health pest globally [1]. All mobile stages of bed bugs feed exclusively on blood from various hosts including humans, domesticated animals, birds, and bats for development and growth [2]. This insect is known for causing physical, psychological, and medical complications in humans [3,4,5]. Several studies had reported that bed bugs may act as competent vectors for various pathogens, including *Bartonella quintana* and *Trypanosoma cruzi*, that cause trench fever and Chagas disease, respectively [4,6,7]. However, direct evidence for disease transmission to humans is still lacking.

Bed bugs were largely eradicated in most parts of the world during the post-World War II era due to wide scale use of pesticides, especially DDT and malathion [8]. Nevertheless, during the late 1990’s, bed bugs made a comeback and their infestations have been rapidly spreading globally [1]. Few theories have been suggested in this regard including the increased rate in global travel and insecticide resistance [9,10]. Some of the bed bugs could have survived in pockets of developing nations where access to insecticides can be limited [1]. The eradication of bed bugs is expensive due to current challenges in control strategies such as insecticide resistance, and this may lead to further spreading of bed bugs globally.

Two species of bed bugs are now closely associated with humans, which are *Cimex lectularius* (L.) and *Cimex hemipterus* (F.). The common bed bug, *C. lectularius*, is now predominantly found in the temperate region, whereas *C. hemipterus* is more common in tropical and subtropical countries. In particular, the growing number of *C. hemipterus* infestations in Southeast Asia has gained public attention in the recent decade [11,12,13,14]. Moreover, the two species can now be found coexisting in several parts of the world including Africa [15], Australia [16], Taiwan [17], Florida [18], and Japan [19] indicating that preconditioning or acclimatization may result in the overlapping of their geographical distributions. The increasing wide spread of bed bug infestations demands an understanding of their population biology and infestation dynamics.

To date, studies on the population genetics and breeding strategy of the bed bugs were heavily focused on the common bed bug, *C. lectularius* [20,21,22,23,24,25], in which these aspects are important to understand the infestation dynamics of this insect. This information, however, remains limited for the tropical species, *C. hemipterus* [26]. Several studies have shown that the two species can be significantly different in terms of their biology and physiology [27,28,29,30], thus an in-depth understanding on their population biology, especially the lesser known *C. hemipterus,* is necessary. Microsatellite markers are especially sensitive for assessing the population genetics of a target species at the molecular level due to their high level of polymorphism, reproducibility, and codominance inheritance. The advent of next-generation sequencing enables the development of microsatellite markers from the genome of non-model organisms such as bed bugs with relative ease [31]. To this extent, this study documents the development and characterization of microsatellite markers from the tropical bed bug *C. hemipterus* for downstream population genetics analysis. We then investigated the infestation dynamics of *C. hemipterus* by understanding the genetic diversity and population structure of *C. hemipterus* in Malaysia and Singapore based on the developed microsatellite markers through fragment analysis. In addition, genetic variations of mtDNA were also assessed based on the partial sequences of 16S rRNA and cytochrome c oxidase I (COI) genes that are widely used for population genetic assessment [32] to offer a greater understanding on the population dynamics of this invasive species. 

## 2. Materials and Methods

### 2.1. Bed Bugs Collection 

**Figure 1:**
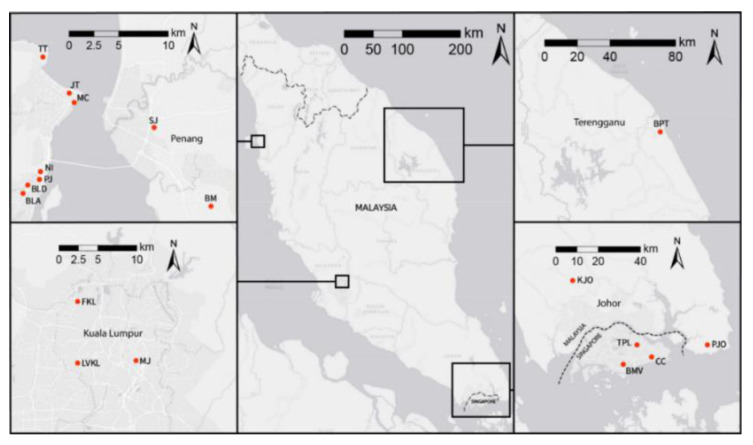
Sampling location of the bed bugs samples from the current study.

*Cimex hemipterus* samples were collected from 18 infested units from Malaysia and Singapore, which includes various types of premises including hostels, hotels, and private residences (Table 1 and Figure 1). We regarded each infestation unit as one population. A total of 351 bed bugs from 18 populations in Malaysia and Singapore were used for the microsatellite analysis. For mtDNA analysis, we included five individuals from each of the 18 populations for a total of 90 samples (Table 1).

### 2.2. 454 Pyrosequencing 

Total genomic DNA was extracted from the whole body of 5th instar nymph using CTB Tissue Extraction Kit according to the manufacturer’s protocol (Intron, Seongnam-Si, Gyeonggi-do, Korea) after being pulverized in liquid nitrogen. DNA quality was assessed by spectrophotometric absorbance in NanoDrop 2000 Spectrophotometer V1.0 (Thermo Fisher Scientific, Wilmington, DE, USA) and Qubit dsDNA BR Assay, followed by electrophoresis on 0.8% agarose gel. In total, ≈2 μg of gDNA extracted from a pool of 15 bed bugs nymphs was used for 1/16th of a 70-mm × 75-mm Titanium PicoTiter plate of sequencing on a Roche 454 GS FLX sequencer with titanium chemistry (Roche Applied Science, Penzberg, Germany), performed by Macrogen Inc., South Korea. Sample preparation and analytical processing of sequence reads were performed according to manufacturer’s protocol for the Titanium series.

### 2.3. Microsatellite Detection and Primer Design

The raw contigs data were processed using Msatcommander v0.8.2 [33]. The motifs of repeat array were screened separately. The repeats lengths were set ≥8, ≥6, ≥5 for dinucleotides, trinucleotides, and tetranucleotides respectively. Primers were designed using Primer3 [34] following Veera Singham et al. [35] by considering amplified regions within a range of 100–500 bp, an optimal TM of 60.0 °C (range 57.0–62.0 °C), possess no greater than 5.0 °C difference in TM, optimal condition of 50% GC content, have at least a 1-bp GC clamp, low levels of self or pair complementarity and a maximum end stability (ΔG) of 8.0.

### 2.4. Characterization of Microsatellite Markers and Genotyping

Forty primer pairs were selected for preliminary tests of polymorphism across ten randomly selected bed bugs from different localities in Malaysia and Singapore. The primer pairs which produced unambiguous PCR products on 1% agarose gel were then evaluated on 6% ethidium bromide-stained polyacrylamide gel electrophoresis (PAGE). Primer pairs that produced scorable, unambiguous PCR products of the expected size, and that demonstrated allelic variation (i.e., at least 3 alleles) on PAGE were selected for further testing. The resulting polymorphic loci were genotyped across a total of 351 bed bugs from 18 populations (see Table 1) by using forward or reverse primers labelled with the fluorescent dyes 6-FAM, HEX, TAMRA-S, and ROX performed in a multiplex PCR containing 12.5 µL total volumes each containing 1X KAPA HiFi Hotstart Readymix (Kapa Biosystems, Wilmington, Massachusetts), primer concentration varied between 0.1 and 0.2 µM, ≈20 ng of DNA template and sterile distilled water up to 12.5 µL. The PCR cycling conditions were set according to Veera Singham et al. [35]. Amplified PCR products were subjected to fragment analysis by electrophoresing on a 3130 Genetic Analyzer (Applied Biosystems, Inc., Foster City, CA, USA). The results of fragment analysis were scored using Peak Scanner version 1.0 (Applied Biosystems, Inc., Foster City, CA, USA) for allelic size determination using GS500 (−250) as an internal size standard.

### 2.5. Microsatellite Data Analyses 

We used the software Arlequin v3.01 [36] to determine departures from Hardy–Weinberg equilibrium, observed and expected heterozygosity (*H*_O_ and *H*_E_), allelic diversity, gene diversity, number of alleles per locus, number of polymorphic loci and linkage disequilibrium among all pairs of loci. Evidence for large allelic dropout, scoring error because of stutter, and null alleles was determined using Microchecker v2.2.3 [37]. The population genetic diversity assessment based on pairwise genetic differentiation (*F*_ST_) and levels of inbreeding (*F*_IS_) in each population were evaluated using FSTAT v2.9.3.2 [38]. Average genetic relatedness (*r*) [39] was estimated using the program Coancestry v1.0.1.7 [40]. The detection of population bottlenecks was estimated using the statistic of Garza–Williamson (G–W) index in Arlequin v3.1 [36]. Partitioning of genetic diversity was evaluated through an analysis of molecular variance (AMOVA) in Arlequin v3.01 at α = 0.05. A Bayesian cluster analysis of population groupings was performed using Bayesian Analysis of Population Structure (BAPS 6.0) [41]. The aim of this analysis is to identify the optimum number of genetic clusters among sample groups and the level of admixture among the different clusters. We used the “Clustering of Individuals” option with an upper bound of 18 populations. The default settings were used following the method by Vargo et al. [42] to identify the proportion of individuals within a cluster showing significant evidence of admixture using the “nonequilbrium” method of individual-based admixture as implemented in BAPS. A posterior probability of 0.05 was set as a critical value for admixture. Isolation-by-distance was tested via Mantel test with 1,000 permutations at α = 0.05 in XLSTAT v2015.2 [43]. The allele frequency distribution for mode shift that discriminates a recently bottlenecked from a stable population was determined according to Luikart et al. [44]. 

### 2.6. Mitochondrial DNA Analyses

A total of 90 bed bugs were subjected to mtDNA analysis targeting the COI and 16S rRNA regions. PCR was conducted using a newly designed internal forward primer CH-COI-F (5′-GGCAGGGATGCTGGGAAC-3′) and the reverse primer LepR (5′-TAWACTTCWGGRTGTCCRAARAATCA-3′) [45] for COI gene, while the primer pair LR-J-13007 (‘5-TTACGCTGTTATCCCTAA-3′) [46] and LR-N-13398 (5′CGCCTGTTTATCAAAAACAT-3′) [47] were used for the 16S rRNA gene using PCR conditions as described in Veera Singham et al. [48]. The PCR amplification was performed in 25 μL reaction containing 1X KAPA HiFi Hotstart Readymix (12.5 µL) (Kapa Biosystems, Wilmington, Massachusetts), each forward and reverse primers at a concentration of 0.2 µM (0.5 µL), ≈20 ng of DNA template (1.0 µL) and sterile distilled water up to 25 µL. Purified PCR products were then subjected to bidirectional Sanger sequencing via sequencing service provider (Apical Scientific, Selangor, Malaysia). Sequence alignment was performed using the ClustalW function in MEGA v.6.0 [49] under default parameters. A partition homogeneity or incongruence length difference (ILD) [50] test as implemented in PAUP^*^ 4.10b [51] was performed to determine if different partitions of the sequence data (i.e., COI and 16S rRNA gene sequences) have significantly different phylogenetic signals. The ILD test was performed with 1000 replicates including 10 random taxon additions per ILD replicate and at a significance level of 0.05. Mitochondrial haplotypes and the diversity indices for the concatenated dataset (aligned in MEGA v.6.0) were then summarized using DnaSP v6.12 [52]. A neighbour-joining (NJ) tree was constructed in MEGA v.6.0 [49] with 10,000 bootstrap replicates following the Kimura 2-parameter model. A maximum likelihood (ML) tree for the concatenated dataset was performed in MEGA v.6.0 [49] implementing the best-fit Hasegawa–Kishino–Yano (HKY) nucleotide substitution model as determined by jModelTest 2.1.10 [53] based on the Akaike information criteria (−lnL = 1263.8923). The ML analysis was conducted following the nearest-neighbor-interchange heuristic method with 1000 bootstrap replicates. Genbank sequences of *C. lectularius* were used as the outgroup: *C. lectularius* 1 (KJ937992.1 for COI and KJ937977.1 for 16S rRNA) and *C. lectularius* 2 (KJ937991.1 for COI and KJ937976.1 for 16S rRNA). The pairwise genetic distance among the haplotypes was also estimated in MEGA v.6.0 using the Kimura-2-parameter model. A median-joining haplotype network was generated using the software Network v10.0 [54] under default settings. Representative nucleotide sequences generated in this study were deposited in the GenBank database under the accession numbers: MT361349–MT361355 for COI gene and MT785743–MT785749 for 16S rRNA gene).

## 3. Results

### 3.1. Development and Characterization of Microsatellite Markers

A modest sequencing volume generated 52,119 reads with 1,821 (3.49%) reads containing di-, tri-, and tetra-nucleotide repeat motifs, with an average length of 242 bp. The search using Msatcommander yielded 620 sequences suitable for primer design consisting of 507 di- (81.77%), 77 tri- (12.42%) and 36 tetra-nucleotide (5.81%) which contain tandem repeats of at least eight, six, and five respectively. Of these primer pairs suitable for primer design, 40 primer pairs were developed successfully (24 di-, 12 tri- and 4 tetra-nucleotides) by using the stringent criteria set in Primer3. Despite having 40 unique microsatellite loci initially, only 23 loci produced unambiguous PCR product (i.e., a single size PCR amplicon within the expected product size on the agarose gel) and were subsequently screened for polymorphism using PAGE. A polymorphic locus is interpreted as a locus that has more than one allele (length variation of microsatellite repeated at a particular locus) found across the individuals tested within or across populations. A locus that exhibited a high number of alleles is more preferred since they have higher discriminating power in examining genetic differences within and between populations. Under this condition, eight loci were found to be polymorphic and consistent in their allelic profile and were therefore subsequently used for downstream population genetic analyses (Table 2). A list of the remaining 15 primers that produced unambiguous PCR products is given in Supplementary Table S1 for future reference. Microchecker analysis showed that no loci exhibited the genetic signature of null alleles, large allelic dropout, or scoring error due to stutter bands. No significant evidence of linkage disequilibrium was detected after the Bonferroni correction (*p* > 0.0028). The mean polymorphic information content (PIC) across loci was 0.54, indicating highly polymorphic markers for downstream population genetic analysis (Table 2).

### 3.2. Population Genetics Analyses Based on the Microsatellite Markers

A total of 351 individuals from 18 populations were genotyped at the eight microsatellite loci. Across all populations, a relatively high genetic diversity was observed with the number of alleles per locus ranging from 4 to 12 alleles (Table 2). The observed and expected heterozygosity for each locus across all populations studied ranged from 0.07 to 0.56 and 0.16 to 0.64, respectively (Table 2). The observed heterozygosities were only slightly lower than expected heterozygosities at all loci except locus Bhe22, which also displayed the lowest number of alleles across the study populations (Table 2). Within each population, several loci were found to deviate significantly from the Hardy–Weinberg equilibrium (HWE) after Bonferroni corrections (Table 3). Nonetheless, the proportion and association of the loci deviating from HWE across the populations were inconsistent, which could be the result of a localized demographic effect such as inbreeding or bottlenecks. Since the markers displayed no evidence of linkage disequilibrium between any pairs of loci (*p* > 0.05) and there was no consistent deviation from HWE for any locus, we regarded the microsatellite markers to be unlinked and therefore suitable for genetic analysis of the study populations. 

Analysis within each populations showed that the observed and expected heterozygosities averaged across all loci ranged from 0.23 to 0.71 and 0.38 to 0.62, respectively, and did not deviate significantly from each other except one population, SJ, that displayed significant heterozygosity deficit (two-tailed *t*-test; *p* < 0.05, Table 3). In terms of allelic diversity, the mean number of alleles per locus for each population ranged from 1.88 to 3.88 alleles (Table 3). We found that 16 of the 18 populations had more than four alleles in at least one of the microsatellite loci (Supplementary Table S2). In fact, seven of these populations had >4 alleles present in at least two loci. 

The Garza–Williamson (G–W) index is a ratio of the number of alleles observed in a sample divided by the number of alleles expected under the observed size range (i.e., number of repeats). Generally, the values of G–W index which is greater than 0.80 are the representative of a stable population that has not suffered a known reduction in size. In the present study, the statistical test indicated a recent bottleneck or founder event in all the studied populations with respect to low G–W index values of less than 0.20 (Table 3). A non-bottlenecked population which is near the mutation–drift equilibrium for selectively neutral loci would have a large proportion of alleles at a low frequency (i.e., <0.1) [44,55,56]. In contrast, a particular population that has been recently bottlenecked is expected to have a pattern of rapid loss of alleles at low frequency [56]. We found that all 18 populations studied showed a lower proportion of alleles at the low frequency than alleles at the intermediate frequency (see Supplementary Figure S1), therefore corroborating the observations from the G–W index.

The coefficient of relatedness (*r*) measures the degree of consanguinity between individuals. We found that the mean coefficient of relatedness in the study populations is 0.373 with a range from 0.124 in BLD to 0.844 in BPT (Table 3). Nearly all of the study populations (n = 14) have the *r* values of between 0.25 and 0.5, which is the value expected for half siblings or grandparent–grandchildren pairs [57] (Table 3). Only one population, BPT, had an *r* value of >0.5, which is expected for siblings and parent/offspring pairs. We also found that the overall inbreeding coefficient, *F*_IS_, across all populations was 0.24 with values ranging from −0.15 to 0.47 and was not significantly greater than zero, indicating that individuals within each infestation unit were not significantly inbred relative to others in the same infestation unit (Table 3). This also shows that the individuals within each infestation unit were randomly mating.

An analysis of molecular variance (AMOVA) indicated a significant level of genetic differentiation at all population levels with variations between populations reported at 28.50% (*p* < 0.001), variations between samples within populations at 18.67% (*p* < 0.001), and the highest variations was within samples at 52.83% (*p* < 0.001) (Table 4). Additionally, we performed AMOVA analysis to investigate the genetic differentiation between bed bug populations from private residential units and public accommodations including hostels and hotels (see Table 1). We found that there was a significant (*p* < 0.05) but low genetic differentiation (2.46%) between the two residential groups whereas genetic differentiation among populations within groups was significantly higher with 26.6% total variance (*p* < 0.05) (Supplementary Table S3). All pairwise *F*_ST_ comparisons between pairs of populations also reported significant genetic differentiation (*p* < 0.05) after Bonferroni corrections, except BLA-SJ (*F*_ST_ = 0.16) (see Supplementary Table S4). This suggests that the populations are genetically isolated and random mating between populations was restricted. The isolation-by-distance analysis revealed no significant correlation between genetic differentiation and geographical distance (*R*^2^ = 0.011; *p* = 0.199) suggesting populations that are geographically proximate to each other are no more similar to each other than populations that are distantly located (Supplementary Figure S2).

Bayesian analysis of population structure (BAPS) is a program for Bayesian inference of the genetic structure in a population whereby the algorithm will attempt to identify the a posteriori most probable partition and calculate the posterior probability for each of them. BAPS analysis returned 17 partitions (clusters) among the 18 study populations, suggesting that each population is genetically isolated, and each made up a unique cluster, except for KJO and PJO which grouped into a single cluster (Table 5). Both of these populations were located in the same state of Johor and the samples were both collected from worker’s hostel; therefore, there is a possibility for bed bugs from these two infestations to be intermixed. Admixture analysis indicated that populations BLD and BLA comprised a significant proportion of admixed individuals although the number is relatively low (Table 5).

### 3.3. MtDNA Analyses

**Figure 2:**
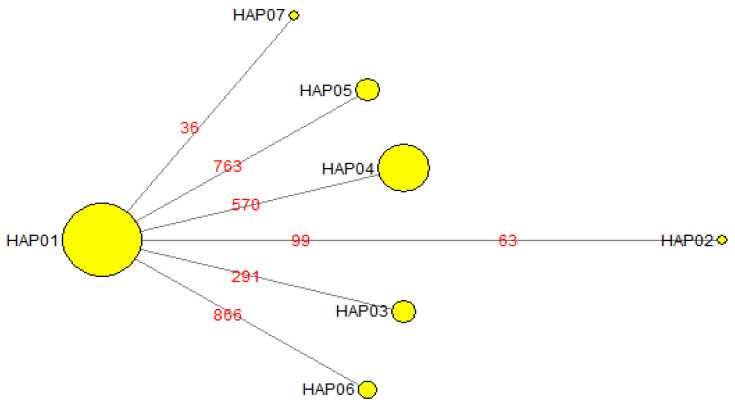
Minimum spanning network showing relationship among seven mtDNA haplotypes based on 909 bp concatenated mtDNA sequences. Each circle denotes inferred haplotypes drawn to proportionate number of individuals. Details of individuals representing each haplotype were given in Table 1. Numbers above branches indicate mutation positions between haplotypes (also see Supplementary Table S5 for information on base positions).

**Figure 3:**
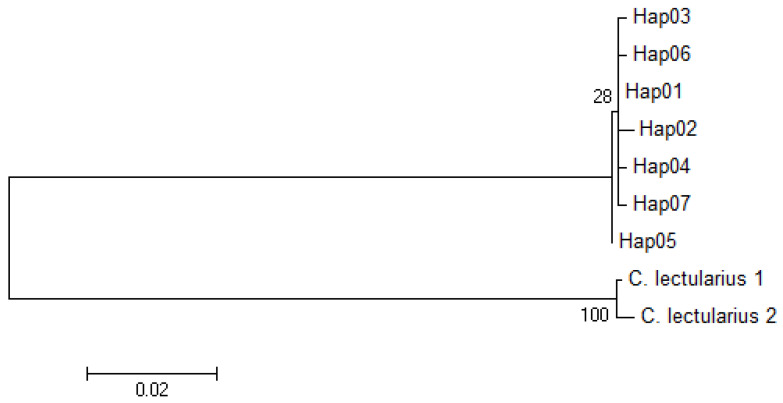
Maximum likelihood phylogenetic tree inferred from seven concatenated 909 bp mtDNA haplotypes. The bootstrap values were provided at each node. Details of individuals representing each haplotype were given in Table 1.

We analysed a total of 90 bed bugs with five individuals each representing the 18 bed bug populations used in microsatellite analysis based on two partial sequences of mtDNA genes. A 575 bp of the COI gene was sequenced and five variable sites were detected. The sequence of the variable sites was confirmed by bidirectional sequencing. Among the five variable sites, two were found parsimony informative. The partial COI sequence was A-T rich with a base composition of Thymine (T) 33.9%, Adenine (A) 29.0%, Guanine (G) 16.9%, and Cytosine (C) 20.2%. For the 16S rRNA gene, only two variable sites were detected, and both were parsimony informative within a 334 bp sequenced region. The nucleotide composition was similarly A-T rich consisting Thymine 43.7%, Adenine 28.7%, Guanine 17.4%, and Cytosine 10.2%. To improve the resolution of the findings, sequences from the two genes were concatenated resulting in a 909 bp aligned sequence. An ILD test resulted in homogeneity between the two gene loci (*p* > 0.05) suggesting the two genes can be concatenated for subsequent downstream analysis. The concatenated dataset resulted in seven haplotypes consisting of seven variable sites, of which four were parsimony informative and with haplotype diversity, Hd = 0.5925 (see Supplementary Table S5 for information on haplotype base positions). Hap01 was the most frequent haplotype represented by 53 individuals, followed by Hap04 with 22 individuals, Hap03 and Hap05 with five individuals, Hap06 with three individuals, whereas Hap02 and Hap07 with one individual each (see Table 1). Five populations, namely NI, TT, PJ, FKL, and LVKL, displayed more than one haplotype within each population with LVKL being the most diverse with three haplotypes (Table 1). Haplotypes Hap03 and Hap05 were each exclusive to two bed bug populations, TPL and BMV from Singapore, respectively, with no haplotype sharing across any other populations. The median-joining minimum spanning network analysis resulted in a starburst pattern with Hap01 forming the central haplotype for all other haplotypes (Figure 2). A maximum likelihood tree was generated to infer the phylogenetic relationship among the bed bug samples from the 18 localities based on the concatenated sequences (Figure 3**)**. No obvious phylogenetic structuring was observed between the haplotypes and the bootstrap support was <70% to significantly support a clade structure [58]. A similar observation was also detected based on the neighbor-joining tree inference (Supplementary Figure S3). Apart from that, the pairwise genetic distances between the haplotypes ranged from 0.001 to 0.003, as would be expected within a species level differentiation (see Supplementary Table S6). Nucleotide diversity, *π*, was 0.00079, and the average number of nucleotide differences, *k*, was 0.718 in the concatenated dataset (Table 6). We detected no signs heteroplasmy (i.e., determined by the presence of any double peaks in the sequence chromatograms) in both the mtDNA genes investigated. 

## 4. Discussion

This study provides an overview on the population genetics and infestation dynamics of the tropical bed bug, *C. hemipterus*, based on two independently inherited molecular markers, namely microsatellites and mtDNA markers. We successfully characterized eight novel polymorphic microsatellite markers that provided insight on the infestation dynamics of *C. hemipterus,* particularly in Malaysia and Singapore. Based on the microsatellite assessments, we found a relatively high genetic diversity across all populations as supported by significant genetic differentiation and distinct genetic structuring observed among the 18 study populations. These observations were consistent with a passive dispersal strategy where interactions between individuals from different populations were restricted, which subsequently led to genetic isolation. The lack of correlation between genetic distance and the physical distance among the sampling units supported this explanation. Although active dispersal is possible in bed bugs [21,59,60,61], passive dispersal remains common in the movement of bed bugs to locate potential hosts [1,21,62,63]. Although our current study did not represent samples that could demonstrate active dispersal as the samples were taken from geographically distant locations (>200 m apart), we found one unique exception. Two sampling units in the state of Johor (Malaysia), KJO and PJO were clustered into a single genetic group (BAPS analysis) suggesting individuals between these two populations were either freely mating or the two populations shared a common source population. Upon further investigation, we discovered that both infested units were worker’s hostels where the occupants were foreign nationals that work in the same food preparation outlet. There is a high likelihood that occupants between these two infested units frequently exchange visits, hence, this increases the chance of bed bugs spreading from one infested unit to another. Alternatively, the bed bugs could have been brought from a common, genetically diverse source population elsewhere (e.g., their country of origin) and subsequently spread to the respective hostels. 

A study by Hentley et al. [64] mentioned that bed bugs were two times more likely to aggregate on bags containing soiled clothes compared to bags containing clean clothes in the absence of a human host. In the present study, most of the field-caught adult *C. hemipterus* in the hotels and hostels were collected from the suitcases and luggage of the occupants, which would explain the spread of *C. hemipterus* by the travellers in this region. Our current sampling populations consist of private residences as well as public accommodations including worker’s hostels, backpacker’s hostels, and hotels. Although significant, the genetic differentiation between the two residential groups was low when compared to genetic differentiation among the populations within each group.

Assessment of allelic diversity within the population level revealed that nearly all the *C. hemipterus* populations in the present study have a high level of allelic diversity (i.e., >4 alleles in at least one locus). This finding is similar with Masran and Majid [26], where all but one of their study populations of *C. hemipterus* displayed >4 alleles in at least one microsatellite locus. This finding could be explained when the foundress of the population was mated to two or more unrelated males and/or their descendants [22]. Another possible scenario could be caused by multiple introduction events of individuals from different source populations or from a common, genetically diverse source population. An exception to this was observed in two populations, NI and KJO, which had ≤4 alleles present at all eight loci. This could be accounted for by the foundress mated to a single male and/or their progeny provided both males and females were heterozygous, resulting in a maximum of four alleles. Alternatively, the population could have been founded by a single female mated to multiple highly-related males. In contrast to *C. hemipterus*, Saenz et al. [22] observed reduced allelic diversity within the common bed bug, *C. lectularius,* populations from the eastern United States whereby 20 of the 21 populations studied had ≤4 alleles across all microsatellite loci, suggesting small propagule size with highly inbred individuals. Similar findings of reduced genetic diversity within aggregations were also found by Booth et al. [21], who studied the infestation dynamics of *C. lectularius* within apartment buildings in Raleigh, NC and Jersey City, NJ, followed by Fountain et al. [25], who investigated *C. lectularius* populations in London. While our study showed a significant reduction of population size as observed by the bottleneck parameters (i.e., G–W index, allele frequency distribution), the allelic diversity suggests that the founding propagule in *C. hemipterus* populations were much diverse and less genetically related than that observed in *C. lectularius* [21,22,25]. This could be either accounted for by the dissimilarities in the natural breeding strategy between the two species or the regional differences of the study populations. Inbreeding in natural populations of animals and plants usually leads to inbreeding depression that causes populations to collapse and increases chances of extinction [65] as a result of the expression of deleterious recessive alleles [66]. It was previously suggested that *C. lectularius* populations are likely able to tolerate high levels of inbreeding or have mechanisms to purge deleterious mutations [21,22]. Such mechanism can be observed in other invasive species such as *Harmonia axyridis* (Pallas) that can purge deleterious alleles that causes inbreeding depression [67]. This condition, however, may not be favoured in the breeding strategy of *C. hemipterus,* as all the populations were not significantly inbred. More studies are therefore needed to understand the impact of inbreeding in both the bed bugs species to affirm these findings. 

Alternatively, local variations due to geographical distribution of the sampling sites could have influenced the observed genetic differences between the two species. For instance, Booth et al. [21] suggested that the *C. lectularius* populations from an apartment building in Raleigh, NC would have a single origin resulting from a sequential founder effect whereas those of Jersey City, NJ apartment buildings would have infested from at least two established lineages as the location of the latter is expected to have high propagule pressure due to the proximity to New York City with a reportedly high level of bed bugs infestations. AMOVA analysis from our study revealed that the highest genetic variations were attributed to individuals in each aggregation more than that observed between populations. Such a scenario suggests that the *C. hemipterus* populations from the present study most likely share a common, genetically diverse source population, and with subsequent isolation and restricted gene flow resulting in higher genetic variations within aggregations. This observation was corroborated by BAPS analysis, which displayed high genetic clustering among the study populations. Two populations (BLA and BLD) also displayed significant admixture which suggests the possibility of further substructure within the aggregations. Masran and Majid’s [26] study on 22 *C. hemipterus* populations in Malaysia reported two genetic clusters based on STRUCTURE analysis. Nonetheless, a significant proportion of the populations studied (nine populations) displayed a mixed membership of both clusters. Evanno et al. [68] indicated caution that delta *K* in STRUCTURE analysis cannot determine the true *K* (i.e., number of clusters) if *K* = 1. The membership to each cluster within the *K* = 2 runs in Masran and Majid [26] was inconsistent with the high levels of admixture suggesting the possibility that a single cluster scenario is more likely. The neighbour-joining tree generated by the authors based on the genetic distance was also poorly resolved to distinguish the two clusters. The authors also reported that samples from all the populations could not be clearly distinguished, as the genetic identity of the bed bugs overlapped in all locations based on their AMOVA and factorial correspondence analysis (FCA), where the former showed a high level of genetic variation within population (83.0%) as observed in the present study. Based on these findings, we can conclude that at the current regional scale, *C. hemipterus* samples collected from Malaysia and Singapore very likely share a common, genetically diverse source population.

Age structure was also thought to be a factor that influences the genetic diversity of a population whereby a more established infestation would favour multi-generational breeding, resulting in low relatedness among the individuals in an aggregation due to random mating, thereby elevating genetic diversity. We found that the overall relatedness was much lower in our study, *r* = 0.373, which was consistent with the findings from Masran and Majid [26] with *r =* 0.174, as opposed to *C. lectularius* with an overall *r* = 0.78 [22], and *r* = 0.437, 0.432, and 0.719 in Raleigh, NC and Jersey City, NJ (JC-A and JC-B), respectively [21]. The mean kinship for *C.* lectularius populations in London was also reportedly high with a value of 0.566 [25]. This could suggest that most of the samples from the present study were collected from units with established *C. hemipterus* infestation except BLD, which has *r* = 0.844, suggesting potential recent introduction. Interestingly, Narain et al. [24] also reported contrasting results from previous reports on *C. lectularius* [21,22,25] whereby they found high heterozygosity and allelic diversity in *C. lectularius* populations from Midwest, USA despite showing high relatedness of >0.5 across of the populations studied, which indicates local variation could have influenced the differences observed.

To further explore the extent of genetic variations in *C. hemipterus*, we investigated the partial DNA sequences of two mtDNA genes, COI and 16S rRNA in understanding. We observed seven haplotypes among the 90 samples examined in the concatenated dataset. Hap01 was the most frequent haplotype that was found in two-thirds of the populations studied and is most likely to be the ancestral haplotype. The starburst pattern of the median-joining haplotype network suggests that all other haplotypes were likely to have derived from haplotype Hap01 with only 1–2 mutational step differences indicating recent expansion (Figure 2; also see Supplementary Table S5). The absence of a significant clade structure in the phylogenetic tree assessment further supports that the bed bug populations in the current study would have originated from a common source population that has recently expanded. We detected that at least five of the populations contain more than one mtDNA haplotype, suggesting multiple introduction events of individuals from different source populations or that the propagules originated from a common, genetically diverse source population, which was consistent with the observations from the microsatellite analyses. The presence of multiple haplotypes within a population is also more common among samples obtained from public accommodations compared to private residential units (i.e., four out of five were hostels), where the chances of multiple introductions can be high due to the frequency of foreign-worker exchange between different locations and new intake of foreign nationals. Interestingly, population NI displayed a maximum of four alleles (Supplementary Table S2) across all microsatellite loci despite having at least two mtDNA haplotypes within the population (Table 1). This finding shows the importance of using two or more independent markers for population genetics assessment. In this case, despite having two mitochondrial lineages, individuals from both lineages displayed similar allelic diversity across the microsatellite loci. In the absence of mtDNA markers, this population would have been thought to have been founded by a single female mated to a single male and/or their progeny, provided both males and females were heterozygous, resulting in a maximum of four alleles, which would underestimate the actual scenario of having more than one matrilineal descent. Nonetheless, the overall mtDNA genetic diversity across all populations is relatively low in comparison to the microsatellite data. Microsatellite loci are considered to generally have a higher mutation rate, often approximately 10^−3^ to 10^−4^ per locus per gamete per generation [69] when compared to mtDNA genes, which would explain the discordance of genetic diversity between the two independently inherited markers. 

In contrast, the mtDNA haplotype diversity and nucleotide diversity from the present study (see Table 6) was much lower than that observed in populations of *C. lectularius* with *h* = 0.861, *π* = 0.006 for 16S rRNA (n = 136; 22 localities) [20] and *h* = 0.7604, *π* = 0.00273 for both COI and 16S rRNA (n = 59; 30 localities) [45]. Szalanski et al. [20] also found a non-significant Tajima’s D statistics and concluded that intermixing, as opposed to a sweep or expansion of haplotypes, had occurred. Findings by Robison et al. [70] later proposed that the elevated mtDNA diversity could be accounted for by the natural occurrence of heteroplasmy in the mtDNA of *C. lectularius* whereby two or more mitochondrial variants were found within a single individual [32]. We found no evidence of heteroplasmy in all of our mtDNA samples for both genes. The complete absence of any overlapping peaks in the mtDNA chromatograms suggests that heteroplasmy is not a common occurrence in *C. hemipterus,* at least within the sampling limit of the current study. However, the PCR-Sanger sequencing approach can fail to detect mitochondrial heteroplasmy as standard sequencing methods may fail to detect the rare mtDNA molecules [71]. Therefore, future studies incorporating a more sensitive allele-specific PCR assay is suggested to confirm the presence of heteroplasmy in *C. hemipterus.*

The reduced mtDNA diversity in *C. hemipterus* populations from the current study could be accounted for by the limitations of the geographical scale and sample size used for the mtDNA analyses in the present study. A more extensive sampling strategy encompassing broader geographical scales is therefore necessary in future studies to further understand the mtDNA inheritance in the tropical bed bug species. Alternatively, the spread of maternally inherited microorganisms, such as *Wolbachia* bacteria, can induce indirect selective sweeps on host mitochondria, resulting in the reduction of effective population size that might lead to smaller mitochondrial diversity [72]. *Wolbachia* is known to be a mutualistic endosymbiont for vitamin B_2_ provisioning in both *C. lectularius* and *C. hemipterus* [73,74]; however, their role in bed bug population biology and reproduction is still limited.

## 5. Conclusions

In conclusion, this study elucidated the infestation dynamics of the tropical bed bug *C. hemipterus* based on two independently inherited molecular markers. The two markers were generally congruent in suggesting a common, genetically diverse (especially at nuclear region) source population for the bed bug samples in the present study. We found a higher overall diversity in nuclear microsatellite markers than mtDNA regions owing to their high level of mutation rate and/or possibly due to selection pressure at the mtDNA regions. Interestingly, we found a contrasting scenario in the population genetic structure and breeding dynamics of the sympatric species, *C. lectularius*. Generally, *C. lectularius* displayed reduced genetic diversity in nuclear DNA (microsatellites and ITS1 region [20]) while displaying elevated diversity in the mtDNA region, though exceptions exist [24]. The discordance in the life strategy and infestation dynamics of the two species was remarkable given the status of the two species being the major cosmopolitan, anthropophilic, and hematophagous members of the Cimicids family. This study therefore demonstrated the importance of understanding the population biology of invasive species, as each species may adopt significantly different strategies for co-existence, especially when competing for the same host. This situation warrants appropriate intervention strategies in managing each bed bug species taking into account the increasing amount of evidence that shows the differences between the two species. The present study is currently limited to a selected region and future studies involving a broader geographical scale are necessary to further understand the intricate population genetics of *C. hemipterus* in its native range. We also recommend the use of multiple independent markers when assessing the population genetics of a target species and findings based on a single marker should be interpreted with caution.

## Figures and Tables

**Table 1:** Samples location and the types of molecular markers used in the study.

Population ID	Longitude	Latitude	Type of Infestation Unit	City, Country	mtDNA Haplotypes ^a^
BMV	1°17′04″ N	103°49′23″ E	Private residence	Bukit Merah, Singapore	Hap05 (5)
TPL	1°20′08″ N	103°51′12″ E	Private residence	Toa Payoh, Singapore	Hap03 (5)
CC	1°19′39″ N	103°55′37″ E	Private residence	Bedok, Singapore	Hap01 (5)
MJ	3°09’05.7″ N	101°41’46.5″ E	Hotel	Jalan Melayu, Kuala Lumpur, Malaysia	Hap01 (5)
LVKL	3°08’41.6″ N	101°41’51.5″ E	Backpacker’s hostel	Jalan Petaling, Kuala Lumpur, Malaysia	Hap01 (2), Hap02 (1), Hap04 (2)
FKL	3°08’39.6″ N	101°41’44.7″ E	Backpacker’s hostel	Jalan Hang Kasturi, Kuala Lumpur, Malaysia	Hap01 (2), Hap06 (3)
BLD	5°19’57.3″ N	100°17’39.8″ E	Worker’s hostel	Bayan Lepas, Penang, Malaysia	Hap01 (5)
TT	5°27’2″ N	100°18’24″ E	Private residence	Tanjung Tokong, Penang, Malaysia	Hap01 (4), Hap07 (1)
BLA	5°19’49″ N	100°17’36″ E	Private residence	Bayan Lepas, Penang, Malaysia	Hap04 (5)
JT	5°25’04.7″ N	100°19’46.0″ E	Worker’s hostel	Georgetown, Penang, Malaysia	Hap01 (5)
NI	5°20’27.6″ N	100°18’06.7″ E	Worker’s hostel	Bayan Lepas, Penang, Malaysia	Hap01 (2), Hap04 (3)
PJ	5°20’18.1″ N	100°18’09.1″ E	Worker’s hostel	Gelugor, Penang, Malaysia	Hap01 (3), Hap04 (2)
MC	5°24’12.2″ N	100°20’03.4″ E	Private residence	Georgetown, Penang, Malaysia	Hap01 (5)
BM	5°18’48.6″ N	100°27’42.6″ E	Worker’s hostel	Bukit Mertajam, Penang, Malayisa	Hap01 (5)
SJ	5°23’08.4″ N	100°24’21.7″ E	Private residence	Seberang Perai, Penang, Malaysia	Hap04 (5)
BPT	5°13’25.8″ N	103°06’27.8″ E	Private residence	Bukit Payong, Terengganu, Malaysia	Hap04 (5)
KJO	1°39’06.3″ N	103°36’58.4″ E	Worker’s hostel	Kulai, Johor, Malaysia	Hap01 (5)
PJO	1°22’08.5″ N	104°06’25.9″ E	Worker’s hostel	Pengerang, Johor, Malaysia	Hap01 (5)

^a^ Haplotypes designation as determined based on the concatenated dataset of 16S rRNA and COI genes. Numbers in bracket indicate number of individuals carrying the haplotype.

**Table 2:** Characteristics of eight microsatellite loci across 18 populations of *C. hemipterus*.

Locus	Primer Sequences (5′–3′)	Repeat Motif	Locus Size (bp)	*N*_A_	PIC	*H*_E_	*H*_O_
Bhe27	GGGCTGATGAAGAAATATAGCAC GGGTTGGGTAAGTTGTGGC	(CT)^8^	385	6	0.51	0.42	0.32
Bhe14	GGAGTTGTTGGGTTAAGGAGTG TCATTCAGGCGATCAAGCC	(ATT)^10^	162	7	0.74	0.64	0.56
Bhe34	TGGGATGTGCAATGTGACC AATGACAGGCCCGAAGTCC	(ATC)^6^	252	5	0.04	0.16	0.11
Bhe07	GCAGTCAAAGACAGTTAGCC GTTGTGGCGTTGTTACGGG	(AC)^9^	310	6	0.53	0.50	0.44
Bhe38	TCGCCTTACACTTCTCGTAG TTTGCATCCCGCTACCCTG	(AAAT)^5^	384	6	0.55	0.52	0.46
Bhe40	CCTTGCCATATCAGCACGTT TGGTGTAATGAACGACCTCTGG	(AAT)^16^	171	12	0.79	0.56	0.45
Bhe12	AACGGATTGGCCTATGAGC CGCACTTGAATAAACAGCCG	(AG)^13^	315	8	0.81	0.61	0.50
Bhe22	ACTCATTTAGGCTCCAGCAAC TGTATCGCGTAGACCCGC	(CT)^8^	331	4	0.35	0.35	0.07
Mean				6.75	0.54	0.47	0.36

*N*_A_ is total number of alleles detected; PIC is polymorphic information content; *H*_E_ is expected heterozygosity; *H*_O_ is observed heterozygosity.

**Table 3:** Summary statistics of 351 *C. hemipterus* samples from 18 populations genotyped at eight microsatellite loci.

Pop	N	*H*_O_	*H*_E_	HWE	G–W	*F*_IS_	*r* (SEM)	A
JT	20	0.64	0.66	Bhe27	0.12	0.47	0. 361 (0.016)	3.75
BLD	20	0.52	0.55	Bhe22	0.13	0.06	0.124 (0.019)	3.88
NI	17	0.36	0.47	-	0.09	0.23	0.494 (0.018)	2.63
MC	20	0.41	0.49	Bhe22	0.10	0.17	0.390 (0.016)	2.88
BM	20	0.40	0.49	Bhe22	0.13	0.18	0.326 (0.019)	3.88
SJ	17	0.33	0.60	Bhe14, Bhe38, Bhe40	0.10	0.46	0.295 (0.019)	3.13
BLA	20	0.34	0.49	Bhe07, Bhe22	0.10	0.31	0.320 (0.020)	3.13
TT	20	0.29	0.40	Bhe22	0.10	0.27	0.361 (0.020)	3.00
PJ	20	0.35	0.55	Bhe12	0.09	0.37	0.331 (0.019)	2.75
BPT	20	0.35	0.37	-	0.06	0.06	0.844 (0.007)	1.88
FKL	20	0.45	0.52	Bhe22	0.11	0.14	0.226 (0.019)	3.25
MJ	20	0.44	0.55	Bhe07, Bhe22	0.11	0.22	0.231 (0.017)	3.25
LVKL	20	0.36	0.42	-	0.10	0.13	0.495 (0.017)	3.13
KJO	20	0.23	0.38	Bhe40, Bhe12	0.09	0.40	0.358 (0.020)	2.63
PJO	17	0.29	0.45	-	0.09	0.36	0.342 (0.019)	2.63
TPL	20	0.27	0.44	Bhe14, Bhe22	0.10	0.39	0.444 (0.020)	3.13
CC	20	0.30	0.41	Bhe07, Bhe38	0.10	0.28	0.495 (0.017)	3.13
BMV	20	0.71	0.62	Bhe22	0.11	−0.15	0.275 (0.019)	3.25
Mean	19.5	0.39	0.49	-	0.10	0.24	0.373 (0.018)	3.07

N is sample size; *H*_E_ is expected heterozygosity; *H*_O_ is observed heterozygosity; HWE refers to locus that shows significant departure from Hardy–Weinberg equilibrium within each population; G–W is Garza–Williamson Index *F*_IS_ is inbreeding coefficient; *r* is relatedness coefficient; A is mean number of alleles per locus.

**Table 4:** Analysis of molecular variance (AMOVA) of *C. hemipterus* as inferred from eight microsatellite markers.

Source of Variation	df	Sum of Square	Variance Component	Total Variance (%)	Fixation Index	*p*-Value
Among populations	17	740.65	1.03	28.50	*F*_ST_ = 0.28	<0.001
Among individuals within populations	333	1088.86	0.68	18.67	*F*_IS_ = 0.26	<0.001
Within individuals	351	672.50	1.92	52.83	*F*_IT_ = 0.46	<0.001

**Table 5:** Bayesian analysis of population structure (BAPS) analysis showing population clusters and the proportion of admixed individuals in each population.

Cluster	Infestation Unit	Proportion of Admixture (Total Sample)
1	BMV	0 (20)
2	TPL	0 (20)
3	CC	0 (20)
4	MJ	0.05 (20)
5	LVKL	0 (20)
6	FKL	0 (20)
7	BLD	0.05 (20) *
8	TT	0 (20)
9	BLA	0.10 (20) *
10	JT	0 (20)
11	NI	0 (17)
12	PJ	0 (20)
13	MC	0 (20)
14	BM	0 (20)
15	SJ	0 (17)
16	BPT	0 (20)
17	KJO	0 (20)
	PJO	0 (17)

* significant admixture; *p* < 0.05.

**Table 6:** Summary statistics of mtDNA genetic variations.

mtDNA Sequence	n	h	Hd	*π* (*k*)	*θ*_s_	*θ*_g_
COI	90	5	0.483 ± 0.049	0.00095 (0.546)	0.00171	0.986
16S rRNA	90	3	0.168 ± 0.052	0.00051 (0.171)	0.00118	0.394
COI + 16S rRNA	90	7	0.593 ± 0.046	0.00079 (0.718)	0.00152	1.380

n is number of sequences, h is number of haplotypes, Hd is haplotype diversity ± SD, *π* is nucleotide diversity, k is average number of nucleotide differences, *θ*_s_ is theta per site, *θ*_g_ is theta per sequence.

## Supplementary Materials

The following are available online at https://www.mdpi.com/2075-4450/11/8/472/s1, Figure S1: Allele frequency distribution for studied populations across eight microsatellite loci. Figure S2: Scatter-plot Mantel test analysis showing the relationship between pairwise *F*_ST_ and geographical distance in km among 18 populations of *C. hemipterus*. Figure S3: Neighbor-joining phylogenetic tree inferred from seven concatenated 909 bp mtDNA haplotypes. Table S1: List of 15 additional primers that produced unambiguous PCR products. Table S2: Number of alleles per population for each microsatellite locus across the 18 populations. Table S3: Analysis of molecular variance (AMOVA) between two residential groups of *C. hemipterus* (public accommodations vs private residential units). Table S4 Pairwise *F*_ST_ and geographic distance between populations of *C. hemipterus*. Supplementary Table S5: Concatenated mtDNA sequences of COI gene (base position 1–575) and 16S rRNA gene (base position 576–909). Table S6: Estimates of evolutionary divergence between haplotypes sequences from concatenated dataset of COI and 16S rRNA genes.

## References

[1] Doggett S.L., Miller D.M., Lee C.Y., Doggett S.L., Miller D.M., Lee C.Y. (2018). Introduction. Advances in the Biology and Management of Modern Bed Bugs.

[2] Usinger R.L. (1966). Monograph of Cimicidae (Hemiptera-Heteroptera).

[3] Hwang S., Svoboda W.T.J., de Jong I.J., Kabasele K.J., Gogosis E. (2005). Bed bug infestations in an urban environment. Emerg. Infect. Dis..

[4] Delaunay P., Blanc V., Giudice P.D., Levy-Becheton A., Chosidow O., Marty P., Brouqui P. (2011). Bedbugs and infectious diseases. Clin. Infect. Dis..

[5] Doggett S.L., Dwyer D.E., Penas P.F., Russell R.C. (2012). Bed bugs: Clinical relevance and control options. Clin. Microbiol. Rev..

[6] Leulmi H., Bitam I., Berenger J.M., Lepidi H., Rolain J.M., Almeras L., Raoult D., Parola P. (2015). Competence of *Cimex lectularius* bed bugs for the transmission of *Bartonella quintana*, the agent of trench fever. PLoS Negl. Trop. Dis..

[7] Salazar R., Castillo-Neyra R., Tustin A.W., Borrini-Mayori K., Naquira C., Levy M.Z. (2015). Bed bugs (*Cimex lectularius*) as vectors of *Trypanosoma cruzi*. Am. J. Trop. Med. Hyg..

[8] Lee C.Y., Doggett S.L., Miller D.M., Doggett S.L., Miller D.M., Lee C.Y. (2018). Chemical control. Advances in the Biology and Management of Modern Bed Bugs.

[9] Dang K., Doggett S.L., Veera Singham G., Lee C.Y. (2017). Insecticide resistance and resistance mechanisms in bed bugs, *Cimex* spp. (Hemiptera: Cimicidae). Parasit. Vectors..

[10] Romero A., Doggett S.L., Miller D.M., Lee C.Y. (2018). Insecticide resistance. Advances in the Biology and Management of Modern Bed Bugs.

[11] How Y.F., Lee C.Y. (2010). Survey of bed bugs in infested premises in Malaysia and Singapore. J. Vector. Ecol..

[12] Tawatsin A., Thavara U., Chompoosri J., Phusup Y., Jonjang N., Khumsawads C., Bhakdeenuan P., Sawanpanyalert P., Asavadachanukorn P., Mulla M.S. (2011). Insecticide resistance in bedbugs in Thailand and laboratory evaluation of insecticides for the control of *Cimex hemipterus* and *Cimex lectularius* (Hemiptera: Cimicidae). J. Med. Entomol..

[13] Khan H.R., Rahman M.M. (2012). Morphology and biology of the bed bugs, *Cimex hemipterus* (Hemiptera: Cimicidae) in the laboratory. J. Biol. Sci..

[14] Leong X.Y., Kim D.Y., Dang K., Veera Singham G., Doggett S.L., Lee C.Y. (2020). Performance of commercial insecticides formulations against different developmental stages of insecticide-resistant tropical bed bugs (Hemiptera: Cimicidae). J. Econ. Entomol..

[15] Newberry K. (1988). Production of a hybrid between the bedbugs *Cimex hemipterus* and *Cimex lectularius*. Med. Vet. Entomol..

[16] Doggett S.L., Geary M.J., Crowe W.J., Wilson P., Russell R.C. (2003). Has the tropical bed bug, *Cimex hemipterus* (Hemiptera: Cimicidae), invaded Australia?. J. Environ. Health..

[17] Lee C.Y. (2013). Bed Bugs in Asia-Perspective from Southeast Asia.

[18] Campbell B.E.P., Koehler G., Buss L.J., Baldwin R.W. (2016). Recent documentation of the tropical bed bug (Hemiptera: Cimicidae) in Florida since the common bed bug resurgence. Fla. Entomol..

[19] Komatsu N., Shirakawa A., Nakamura H., Fujii K. (2018). Distribution of tropical bedbug *Cimex hemipterus* in Tokyo, Japan. Med. Entomol. Zool..

[20] Szalanski A.L., Austin J.W., McKern J.A., Steelman C.D., Gold R.E. (2008). Mitochondrial and ribosomal internal transcribed spacer 1 diversity of *Cimex lectularius* (Hemiptera: Cimicidae). Genetics.

[21] Booth W., Saenz V.L., Santangelo R.G., Wang C., Schal C., Vargo E.L. (2012). Molecular markers reveal infestation dynamics of the bed bug (Hemiptera: Cimicidae) within apartment buildings. J. Med. Entomol..

[22] Saenz V.L., Booth W., Schal C., Vargo E.L. (2012). Genetic analysis of bed bug populations reveals small propagule size within individual infestations but high genetic diversity across infestations from the Eastern United States. J. Med. Entomol..

[23] Akhoundi M., Kegne P., Cannet A., Brengues C., Berenger J.M., Izri A., Marty P., Simard F., Fontenille D., Delaunay P. (2015). Spatial genetic structure and restricted gene flow in bed bugs (*Cimex lectularius*) populations in France. Infect. Genet. Evol..

[24] Narain R.B., Latihambika S., Ksamble S.T. (2015). Genetic variability and geographic diversity of the common bed bug (Hemiptera: Cimicidae) populations from the Midwest using microsatellite markers. J. Med. Entomol..

[25] Fountain T., Duvaux L., Horsburgh G., Reinhardt K., Butlin R.K. (2014). Human-facilitated metapopulation dynamics in an emerging pest species, *Cimex lectularius*. Mol. Ecol..

[26] Masran S.N.A.S., Majid A.H.A. (2019). Population genetic structure and breeding pattern of *Cimex hemipterus* (F.) (Hemiptera: Cimicidae) in Malaysia. J. Med. Entomol..

[27] Reinhardt K., Siva-Jothy M.T. (2007). Biology of the bed bugs (Cimicidae). Annu. Rev. Entomol..

[28] How Y.F., Lee C.Y. (2010). Effects of temperature and humidity on survival and water loss of *Cimex hemipterus* (Hemiptera: Cimicidae). J. Med. Entomol..

[29] Dang K., Toi C.S., Lilly D.G., Lee C.Y., Naylor R., Tawatsin A., Thavara U., Wu B., Dogget S.L. (2015). Identification of putative *kdr* mutations in the tropical bed bug, *Cimex hemipterus* (Hemiptera: Cimicidae). Pest. Manag. Sci..

[30] Kim D., Billen J., Doggett S.L., Lee C.Y. (2017). Differences in climbing ability between *Cimex lectularius* and *Cimex hemipterus* (Hemiptera: Cimicidae). J. Econ. Entomol..

[31] Gardner M.G., Fitch A.J., Bertozzi T., Lowe A.J. (2011). Rise of the machines—Recommendations for ecologists when using the next generation sequencing for microsatellite development. Mol. Ecol. Resour..

[32] Avise J.C. (2004). Molecular Markers, Natural History and Evolution.

[33] Faircloth B.C. (2008). MSATCOMMANDER: Detection of microsatellite repeat arrays and automated, locus-specific primer design. Mol. Ecol. Resour..

[34] Rozen S., Skaletsky H. (2000). Primer3 on the WWW for general users and for biologist programmers. Methods Mol. Biol..

[35] Veera Singham G., Othman A.S., Vargo E.L., Booth W., Lee C.Y. (2012). Polymorphic microsatellite loci from an indigenous Asian fungus-growing termite, *Macrotermes gilvus* (Blattodea: Termitidae) and cross amplification in related taxa. Environ. Entomol..

[36] Excoffier L., Laval G., Schneider S. (2005). ARLEQUIN ver. 3.01: An integrated software package for population genetics data analysis. Evol. Bioinform..

[37] Van Oosterhout C.V., Hutchinson W.F., Wills D.P.M., Shipley P. (2004). MICRO- CHECKER: Software for identifying and correcting genotyping errors in microsatellite data. Mol. Ecol. Notes.

[38] Goudet J. (1995). FSTAT (Version 1.2): A computer program to computer note computer program to calculate F-statistics. J. Hered..

[39] Queller D.C., Goodnight K.F. (1989). Estimating relatedness using genetic markers. Evolution.

[40] Wang J. (2011). COANCESTRY: A program for simulating, estimating and analyzing relatedness and inbreeding coefficients. Mol. Ecol. Resour..

[41] Corrander J.A., Marttinen P., Siren J., Tang J. (2008). Enhanced Bayesian modelling in BAPS software for learning genetic structure of populations. BMC Bioinform..

[42] Vargo E.L., Crissmann J.R., Booth W., Santangelo R.G., Mukha D.V., Schal C. (2014). Hierarchical genetic analysis of German cockroach (*Blatella germanica*) populations from within buildings to across continents. PLoS ONE.

[43] Addinsoft. XLSTAT-Statistics Package for Excel. https://www.xlstat.com.

[44] Luikart G., Allendorf F.W., Cornuet J.M., Sherwin W.B. (1998). Distortion of allele frequency distributions provides a test for recent population bottlenecks. J. Hered..

[45] Balvin O., Munclinger P., Kratochvil L., Vílímová J. (2012). Mitochondrial DNA and morphology show independent evolutionary histories of bedbug *Cimex lectularius* (Heteroptera: Cimicidae) on bats and humans. Parasitol. Res..

[46] Kambhampati S., Smith P.T. (1995). PCR primers for the amplification of four insect mitochondrial gene fragments. Insect Mol. Biol..

[47] Simon C., Frati F., Beckenbach A., Crespi B., Liu H., Flook P. (1994). Evolution, weighting and phylogenetic utility of mitochondrial gene sequences and a compilation of conserved polymerase chain reaction primers. Ann. Entomol. Soc. Am..

[48] Veera Singham G., Othman A.S., Lee C.Y. (2017). Phylogeography of the termite *Macrotermes gilvus* and insight into ancient dispersal corridors in Pleistocene Southeast Asia. PLoS ONE.

[49] Tamura K., Stecher G., Peterson D., Filipski A., Kumar S. (2013). MEGA6: Molecular evolutionary genetics analysis version 6.0. Mol. Biol. Evol..

[50] Farris J.S., Källersjö M., Kluge A.G., Bult C. (1994). Testing significance of incongruence. Cladistics.

[51] Swofford D.L. (2003). PAUP*. Phylogenetic Analysis Using Parsimony and Other Methods Version 4.

[52] Rozas J., Ferrer-Mata A., Sánchez-DelBarrio J.C., Guirao-Rico S., Librado P., Ramos-Onsins S.E., Sánches-Gracia A. (2017). DnaSP 6: DNA sequence polymorphism analysis of large datasets. Mol. Biol. Evol..

[53] Darriba D., Taboada G.L., Doallo R., Posada D. (2012). jModelTest 2: More models, new heuristics and parallel computing. Nat. Methods.

[54] Bandelt H.J., Foster P., Rohl A. (1999). Median-joining networks for inferring intraspecific phylogenies. Mol. Biol. Evol..

[55] Chakraborty R., Fuerst P.A., Nei M. (1980). Statistical studies on protein polymorphism in natural populations III. Distribution of allele frequencies and the number of alleles per locus. Genetics.

[56] Nei M., Maruyama T., Chakraborty R. (1975). The bottleneck effect and genetic variability in populations. Evolution.

[57] Taylor H.R. (2015). The use and abuse of genetic marker-based estimates of relatedness and inbreeding. Ecol. Evol..

[58] Hillis D.M., Bull J.J. (1993). An empirical test of bootstrapping as a method for assessing confidence in phylogenetic analysis. Syst. Biol..

[59] Mellanby K. (1939). The physiology and activity of the bedbug (*Cimex letularius* L.) in a natural infestation. J. Parasitol..

[60] Pfiester M., Koehler P.G., Pereira R.M. (2009). Sexual conflict to the extreme: Traumatic insemination in bed bugs. Entomol. Am..

[61] How Y.F., Lee C.Y. (2010). Effects of life-stages and feeding regimes on active movement behavior of the tropical bed bug, *Cimex hemipterus* (Hemiptera: Cimicidae). J. Med. Entomol..

[62] Newberry K., Mchunu Z.M., Cebekhulu S.Q. (1991). Bedbug reinfestation rates in rural Africa. Med. Vet. Entomol..

[63] Boase C. (2001). Bedbugs—Back from the brink. Pest Manag. Sci..

[64] Hentley W.T., Webster B., Evison S.F.E., Siva-Jothy M.T. (2017). Bed bug aggregation on dirty laundry: A mechanism for passive dispersal. Sci. Rep..

[65] Keller L.F., Waller D.M. (2002). Inbreeding effects in wild populations. Trends Ecol. Evol..

[66] Charlesworth B., Charlesworth D. (1999). The genetic basis of inbreeding depression. Genet. Res..

[67] Facon B., Hufbauer R.A., Tayeh A., Loiseau A., Lombaert E., Vitalis R., Guillemaud T., Lundgren J.G., Estoup A. (2011). Inbreeding depression is purged by the invasive insect *Harmonia axyridis*. Curr. Biol..

[68] Evanno G., Regnaut S., Goudet J. (2005). Detecting the number of clusters of individuals using the software STRUCTURE: A simulation study. Mol. Ecol..

[69] Primmer C.R., Ellegren H., Saino N., Møller A.P. (1996). Directional evolution in germline microsatellite mutations. Nat. Genet..

[70] Robison G.A., Balvin O., Schal C., Vargo E.L., Booth W. (2015). Extensive mitochondrial heteroplasmy in natural populations of a resurging human pest, the bed bug (Hemiptera: Cimicidae). J. Med. Entomol..

[71] Mastrantonio V., Latrofa M.S., Porretta D., Lia R.P., Parisi A., Latta R., Dantas-Torres F., Otranto D., Urbanelli S. (2019). Paternal leakage and mtDNA heteroplasmy in *Rhipicephalus* spp. ticks. Sci. Rep..

[72] Cariou M., Duret L., Charlat S. (2017). The global impact of *Wolbachia* on mitochondrial diversity and evolution. J. Evol. Biol..

[73] Hosokawa T., Koga R., Kikuchi Y., Meng X.Y., Fukatsu T. (2010). *Wolbachia* as a bacteriocyte-associated nutritional mutualist. Proc. Natl. Acad. Sci. USA.

[74] Balvin O., Roth S., Talbot B., Reindhart K. (2018). Co-speciation in bedbug Wolbachia parallel the pattern in nematode host. Sci. Rep..

