# Supplementary material for: Infestation Pattern and Population Dynamics of the Tropical Bed Bug, Cimex hemipterus (F.) (Hemiptera: Cimicidae) Based on Novel Microsatellites and mtDNA Markers
Source: Insects. 2020 Jul 25;11(8):472. doi: 10.3390/insects11080472 (PMC7469168; doi:10.3390/insects11080472)
Supplement: Supplementary file 1 [file insects-11-00472-s001.zip › insects-875826-supplementary_proof_revised/Supplementary Figure S1.docx]

| Number of alleles | 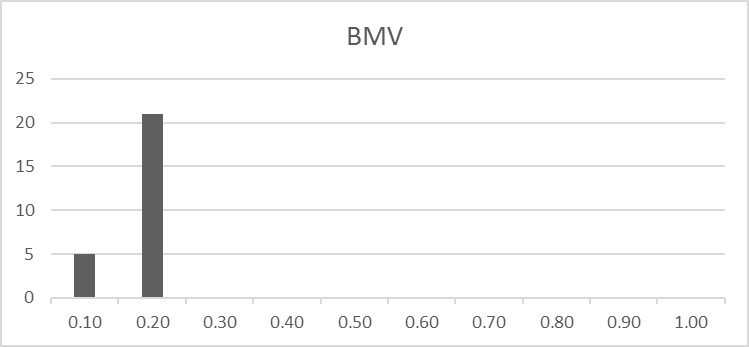 | 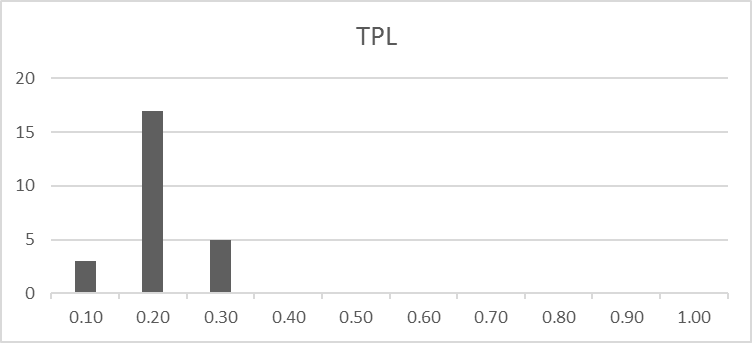 | 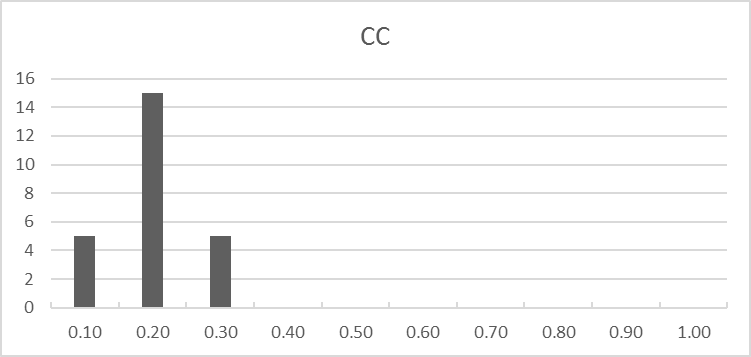 |
| --- | --- | --- | --- |
|  | 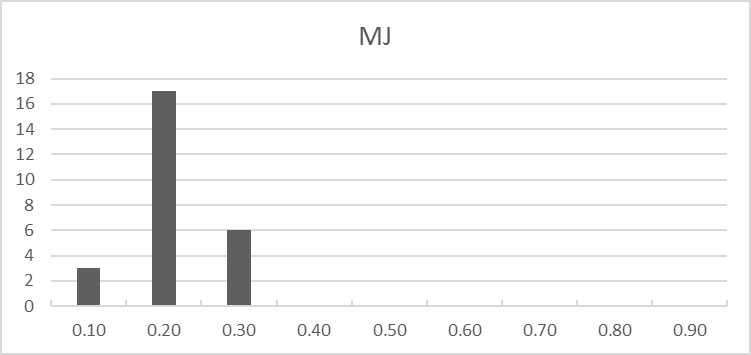 | 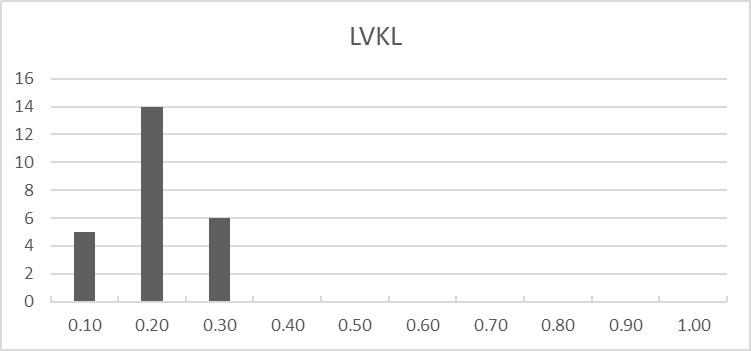 | 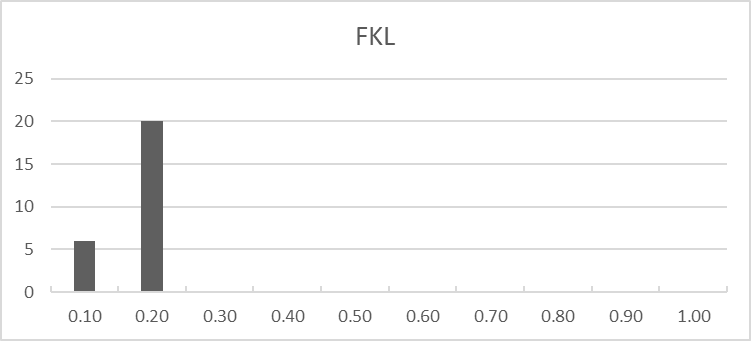 |
|  | 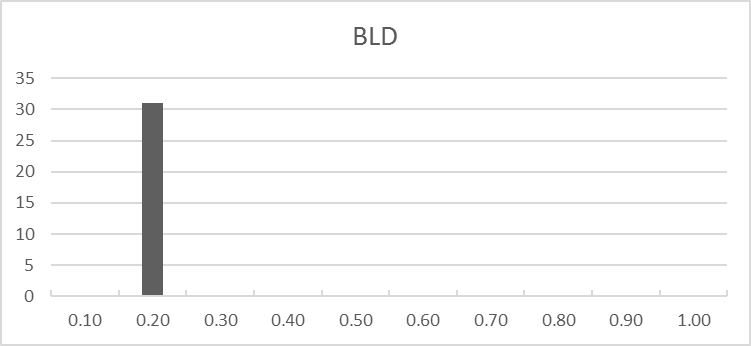 | 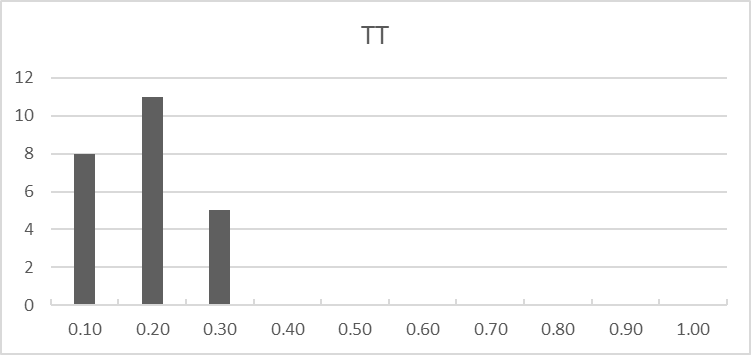 | 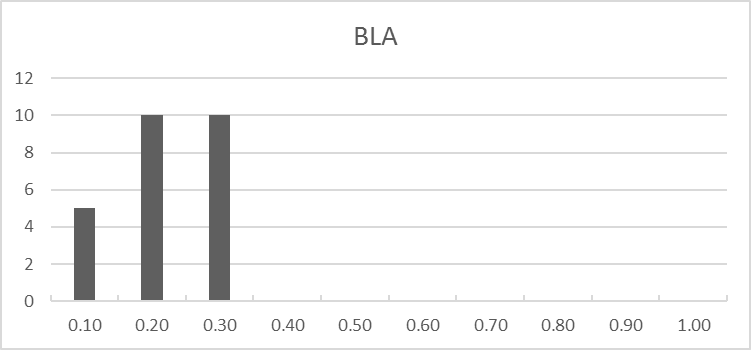 |
|  | Allele frequency class | | |
|  | **Supplementary Figure S1:** Allele frequency distribution for studied populations across eight microsatellite loci. | | |

| 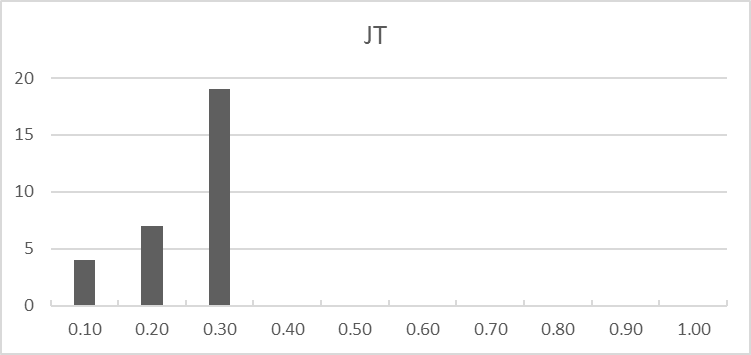  Number of alleles | 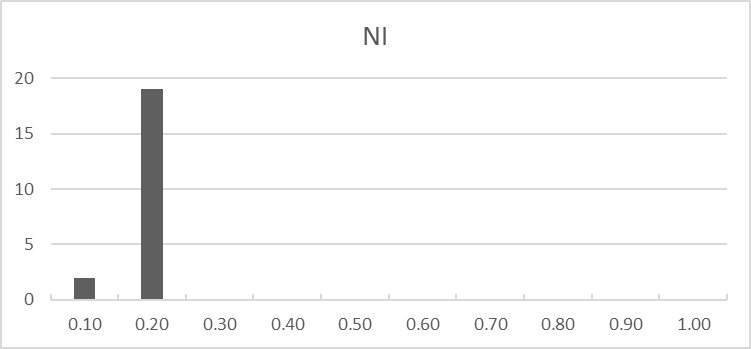 | 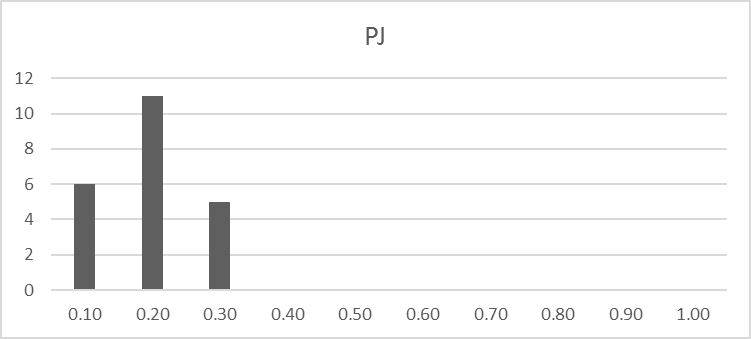 |
| --- | --- | --- |
| 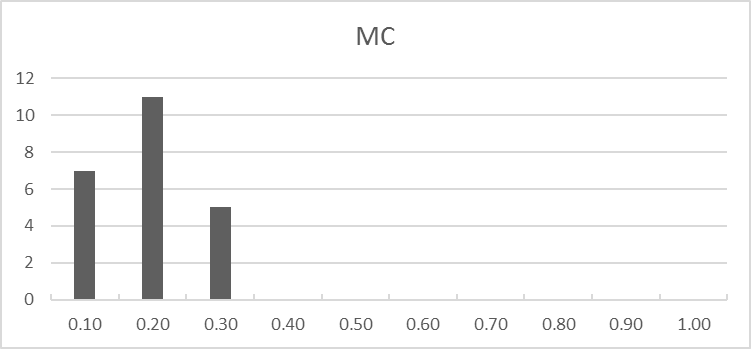 | 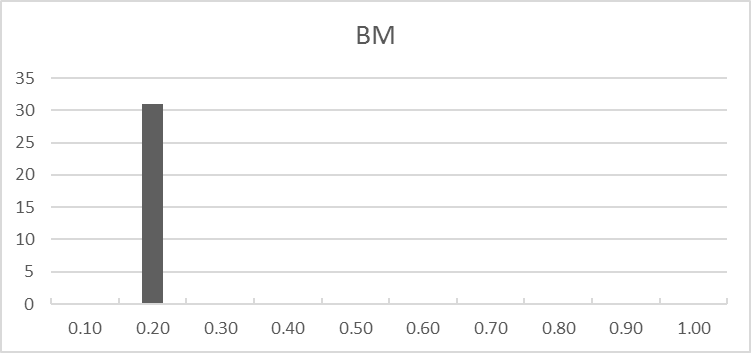 | 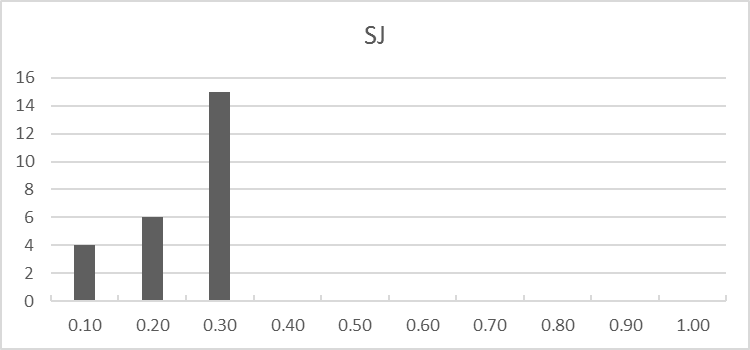 |
| 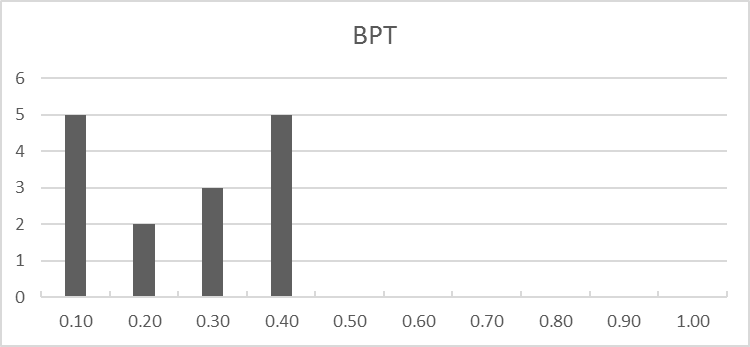 | 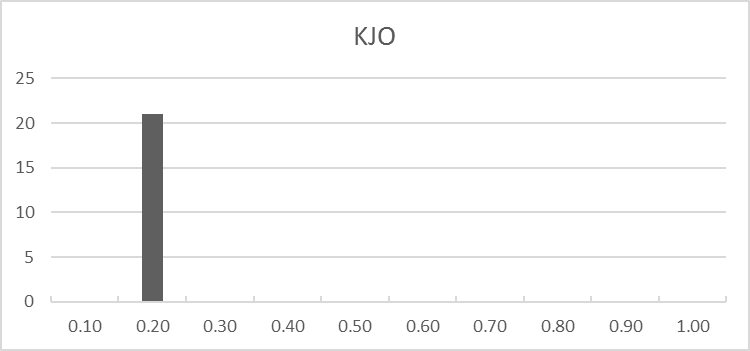 | 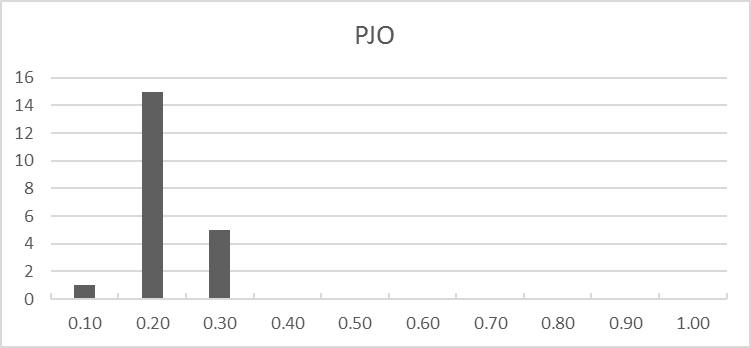 |
| Allele frequency class | | |
| **Supplementary Figure S1:** Continued. | | |
