# Supplementary material for: Infestation Pattern and Population Dynamics of the Tropical Bed Bug, Cimex hemipterus (F.) (Hemiptera: Cimicidae) Based on Novel Microsatellites and mtDNA Markers
Source: Insects. 2020 Jul 25;11(8):472. doi: 10.3390/insects11080472 (PMC7469168; doi:10.3390/insects11080472)
Supplement: Supplementary file 1 [file insects-11-00472-s001.zip › insects-875826-supplementary_proof_revised/Supplementary Figure S3.docx]

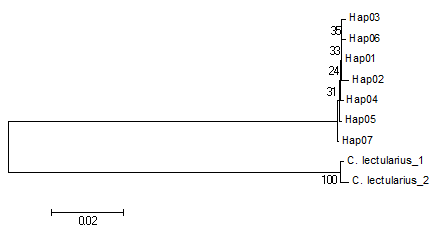


**Supplementary Figure S3.** Neighbor-joining phylogenetic tree inferred from seven concatenated 909 bp mtDNA haplotypes. The bootstrap values were provided at each node.
