# Supplementary material for: Infestation Pattern and Population Dynamics of the Tropical Bed Bug, Cimex hemipterus (F.) (Hemiptera: Cimicidae) Based on Novel Microsatellites and mtDNA Markers
Source: Insects. 2020 Jul 25;11(8):472. doi: 10.3390/insects11080472 (PMC7469168; doi:10.3390/insects11080472)
Supplement: Supplementary file 1 [file insects-11-00472-s001.zip › insects-875826-supplementary_proof_revised/Supplementary Table S1_rev.docx]

| Locus | Primer sequences (5’ – 3’) | Repeat motif | Locus size (bp) |
| --- | --- | --- | --- |
| Bhe 02 | GCTGCAACCGACCAGATATTC  ATTTACCAAAGTGCCCGCTG | (GT)^14 | 326 |
| Bhe 03 | CCAGCATCCCTGCATTCG  GGTCGTTATCCCTCGAGCC | (AT)^10 | 192 |
| Bhe 06 | TTAGGCTCAGTGTTTGCCC  GACGTTCGATGGCTTCAGG | (GT)^14 | 291 |
| Bhe 09 | ACGGGAATACGGGATAAGGC  CCAAATGGCATGAAGCTAGAAAC | (AT)^13 | 402 |
| Bhe 11 | AGTTCCTAGCTGCCGATGG  GTGGGTGGTGGTCCGATAC | (GT)^9 | 228 |
| Bhe 17 | TCTCTGATGATGTCCCTGAAAG  TATCGCTTGGCTCAATGCC | (ATT)^6 | 315 |
| Bhe 21 | CTCAAATGCATGGCCTCTGG  CTTGGCGGTAAACTGCGAC | (AT)^8 | 429 |
| Bhe 23 | TTTACAGCGAGTCCCAAGC  AATTATTCCGACCGGCACG | (AC)^9 | 398 |
| Bhe 24 | TGCCTATTGCTGTCTCCAG  TCGCACCCATTGTTAAATCAGG | (AT)^8 | 311 |
| Bhe 25 | GACCGCAATGTCACGCTTC  TTGGGCTCAGACGATAGATCC | (AT)^9 | 169 |
| Bhe 26 | CCACTGTGACCAGGGAC  ATTTCGCTGTACGCACTTCC | (AT)^10 | 379 |
| Bhe 29 | TGCAGGTATTCGCTCGTAG  ATGTGTTCAGTCCAGTAGGG | (AG)^15 | 346 |
| Bhe 31 | GGCGGCCTTTATGAAATGGG  CTCCCTGATTCGTTGCCAG | (AG)^12 | 246 |
| Bhe 36 | CATGAGTAGGACACGATTGC  CATATTCGGACGGGAAGAAACC | (GAT)^7 | 352 |
| Bhe 37 | ATGCTGCTCCCACCACTTG  ACCTGATCATTCGAGAGACCC | (ATTT)^5 | 418 |

**Supplementary Table S1:** List of 15 additional primers that produced unambiguous PCR products.
