# Supplementary material for: Infestation Pattern and Population Dynamics of the Tropical Bed Bug, Cimex hemipterus (F.) (Hemiptera: Cimicidae) Based on Novel Microsatellites and mtDNA Markers
Source: Insects. 2020 Jul 25;11(8):472. doi: 10.3390/insects11080472 (PMC7469168; doi:10.3390/insects11080472)
Supplement: Supplementary file 1 [file insects-11-00472-s001.zip › insects-875826-supplementary_proof_revised/Supplementary Table S2_rev.docx]

**Supplementary Table S2**: Number of alleles per population for each microsatellite locus across the 18 populations.

| Populations | | | | | | | | | | | | | | | | | | | |
| --- | --- | --- | --- | --- | --- | --- | --- | --- | --- | --- | --- | --- | --- | --- | --- | --- | --- | --- | --- |
|  | JT | BLD | MC | BM | SJ | BLA | NI | TT | PJ | BPT | FKL | MJ | LVKL | KJO | PJO | TPL | CC | BMV | Total |
| Locus/N | 20 | 20 | 20 | 20 | 17 | 20 | 17 | 20 | 20 | 20 | 20 | 20 | 20 | 20 | 17 | 20 | 20 | 20 | 351 |
| Bhe27 | 4 | 3 | 3 | 5 | 3 | 2 | 2 | 2 | 2 | 1 | 3 | 3 | 3 | 2 | 2 | 4 | 2 | 4 | 6 |
| Bhe14 | 6 | 6 | 5 | 6 | 5 | 4 | 4 | 3 | 5 | 3 | 5 | 3 | 4 | 3 | 2 | 3 | 5 | 5 | 7 |
| Bhe34 | 1 | 4 | 2 | 3 | 1 | 1 | 1 | 2 | 1 | 1 | 3 | 1 | 1 | 2 | 1 | 1 | 1 | 1 | 5 |
| Bhe07 | 2 | 4 | 4 | 3 | 5 | 3 | 1 | 2 | 2 | 2 | 2 | 4 | 2 | 2 | 3 | 3 | 4 | 2 | 6 |
| Bhe38 | 1 | 3 | 2 | 3 | 3 | 3 | 3 | 4 | 4 | 1 | 2 | 2 | 3 | 2 | 2 | 2 | 4 | 3 | 6 |
| Bhe40 | 7 | 3 | 1 | 4 | 5 | 5 | 4 | 4 | 3 | 1 | 4 | 4 | 6 | 4 | 4 | 4 | 3 | 4 | 12 |
| Bhe12 | 6 | 5 | 4 | 4 | 2 | 5 | 4 | 5 | 4 | 5 | 5 | 6 | 4 | 4 | 5 | 5 | 4 | 5 | 8 |
| Bhe22 | 3 | 3 | 2 | 3 | 1 | 2 | 2 | 2 | 1 | 1 | 2 | 3 | 2 | 2 | 2 | 3 | 2 | 2 | 4 |
| Mean | 3.75 | 3.88 | 2.88 | 3.88 | 3.13 | 3.13 | 2.63 | 3.00 | 2.75 | 1.88 | 3.25 | 3.25 | 3.13 | 2.63 | 2.63 | 3.13 | 3.13 | 3.25 | 3.07 |
| Total | 30 | 31 | 23 | 31 | 25 | 25 | 21 | 24 | 22 | 15 | 26 | 26 | 25 | 21 | 21 | 25 | 25 | 26 | 54 |

N is sample size.
