# Supplementary material for: Infestation Pattern and Population Dynamics of the Tropical Bed Bug, Cimex hemipterus (F.) (Hemiptera: Cimicidae) Based on Novel Microsatellites and mtDNA Markers
Source: Insects. 2020 Jul 25;11(8):472. doi: 10.3390/insects11080472 (PMC7469168; doi:10.3390/insects11080472)
Supplement: Supplementary file 1 [file insects-11-00472-s001.zip › insects-875826-supplementary_proof_revised/Supplementary Table S3_rev.docx]

| Source of variation | df | Sum of square | Variance component | Total variance (%) | Fixation index | *p*-value |
| --- | --- | --- | --- | --- | --- | --- |
| Among groups | 1 | 47.50 | 0.06 | 2.46 | *F*_CT_ = 0.02 | <0.05 |
| Among populations within groups | 16 | 430.44 | 0.64 | 26.57 | *F*_SC_ = 0.27 | <0.05 |
| Among individuals within populations | 333 | 684.17 | 0.35 | 14.68 | *F*_IS_ = 0.21 | <0.05 |
| Within individuals | 351 | 474.00 | 1.35 | 56.29 | *F*_IT_ = 0.44 | <0.05 |

**Supplementary Table S3.** Analysis of molecular variance (AMOVA) between two residential groups of *C. hemipterus* (public accommodations vs private residential units).
