# Supplementary material for: Infestation Pattern and Population Dynamics of the Tropical Bed Bug, Cimex hemipterus (F.) (Hemiptera: Cimicidae) Based on Novel Microsatellites and mtDNA Markers
Source: Insects. 2020 Jul 25;11(8):472. doi: 10.3390/insects11080472 (PMC7469168; doi:10.3390/insects11080472)
Supplement: Supplementary file 1 [file insects-11-00472-s001.zip › insects-875826-supplementary_proof_revised/Supplementary Table S4_rev.docx]

**Supplementary Table S4:** Pairwise *F*_ST_ values between populations of *C*. *hemipterus* (below diagonal) and pairwise geographic distance (km)(above diagonal). All *F*_ST_ values were significant at α = 0.05 level (except in bold) after Bonferroni correction.

|  | JT | BMV | FKL | BLD | MC | BM | MJ | TPL | LVKL | SJ | BLA | CC | NI | TT | BPT | PJ | KJO | PJO |
| --- | --- | --- | --- | --- | --- | --- | --- | --- | --- | --- | --- | --- | --- | --- | --- | --- | --- | --- |
| JT | * | 601.41 | 294.73 | 10.26 | 1.71 | 25.10 | 294.07 | 599.34 | 294.79 | 9.21 | 10.54 | 605.21 | 9.09 | 4.41 | 308.36 | 9.34 | 555.33 | 615.19 |
| BMV | 0.17 | * | 314.14 | 596.74 | 599.82 | 607.42 | 314.63 | 6.51 | 314.02 | 593.20 | 596.62 | 12.54 | 596.90 | 605.8 | 445.29 | 596.63 | 46.97 | 32.99 |
| FKL | 0.12 | 0.16 | * | 288.71 | 293.07 | 297.29 | 0.81 | 313.06 | 0.22 | 287.34 | 288.55 | 319.79 | 289.05 | 299.14 | 279.26 | 288.76 | 270.31 | 332.79 |
| BLD | 0.16 | 0.13 | 0.15 | * | 9.03 | 18.49 | 288.06 | 594.77 | 288.77 | 13.70 | 0.28 | 600.72 | 1.25 | 13.19 | 311.74 | 1.11 | 550.82 | 610.98 |
| MC | 0.23 | 0.24 | 0.25 | 0.18 | * | 24.88 | 292.40 | 597.76 | 293.12 | 8.18 | 9.31 | 603.64 | 7.81 | 6.07 | 307.72 | 8.04 | 553.76 | 613.64 |
| BM | 0.15 | 0.18 | 0.15 | 0.17 | 0.25 | * | 296.66 | 605.60 | 297.36 | 31.76 | 18.34 | 611.69 | 19.44 | 24.93 | 330.03 | 19.46 | 561.77 | 622.38 |
| MJ | 0.17 | 0.17 | 0.13 | 0.14 | 0.19 | 0.12 | * | 313.54 | 0.76 | 286.67 | 287.9 | 320.26 | 288.40 | 298.47 | 278.56 | 288.11 | 270.76 | 333.23 |
| TPL | 0.25 | 0.20 | 0.16 | 0.25 | 0.37 | 0.26 | 0.25 | * | 312.94 | 591.09 | 594.65 | 8.21 | 594.92 | 603.73 | 440.42 | 594.65 | 44.12 | 28.49 |
| LVKL | 0.28 | 0.24 | 0.25 | 0.23 | 0.34 | 0.23 | 0.28 | 0.33 | * | 287.39 | 288.61 | 319.67 | 289.11 | 299.19 | 279.09 | 288.82 | 270.18 | 332.66 |
| SJ | 0.25 | 0.24 | 0.24 | 0.20 | 0.30 | 0.25 | 0.20 | 0.36 | 0.29 | * | 13.91 | 596.92 | 12.56 | 13.15 | 299.67 | 12.61 | 547.06 | 606.79 |
| BLA | 0.25 | 0.25 | 0.25 | 0.19 | 0.25 | 0.23 | 0.20 | 0.32 | 0.32 | **0.16** | * | 600.61 | 1.52 | 13.46 | 311.84 | 1.36 | 550.71 | 610.88 |
| CC | 0.32 | 0.32 | 0.27 | 0.25 | 0.32 | 0.30 | 0.24 | 0.42 | 0.40 | 0.25 | 0.26 | * | 600.86 | 609.59 | 442.69 | 600.59 | 49.93 | 20.56 |
| NI | 0.34 | 0.27 | 0.27 | 0.24 | 0.41 | 0.29 | 0.31 | 0.41 | 0.41 | 0.37 | 0.35 | 0.31 | * | 12.19 | 310.95 | 0.30 | 550.96 | 611.08 |
| TT | 0.26 | 0.19 | 0.20 | 0.16 | 0.25 | 0.23 | 0.12 | 0.33 | 0.32 | 0.24 | 0.19 | 0.25 | 0.28 | * | 311.14 | 12.48 | 559.72 | 619.56 |
| BPT | 0.46 | 0.38 | 0.43 | 0.33 | 0.47 | 0.46 | 0.41 | 0.46 | 0.47 | 0.46 | 0.45 | 0.54 | 0.57 | 0.43 | * | 310.86 | 401.19 | 442.76 |
| PJ | 0.24 | 0.20 | 0.20 | 0.10 | 0.32 | 0.25 | 0.24 | 0.32 | 0.32 | 0.28 | 0.26 | 0.33 | 0.20 | 0.25 | 0.43 | * | 550.69 | 610.82 |
| KJO | 0.26 | 0.28 | 0.26 | 0.22 | 0.34 | 0.27 | 0.28 | 0.50 | 0.32 | 0.33 | 0.29 | 0.36 | 0.36 | 0.31 | 0.50 | 0.19 | * | 62.98 |
| PJO | 0.26 | 0.25 | 0.27 | 0.20 | 0.30 | 0.26 | 0.25 | 0.42 | 0.30 | 0.29 | 0.25 | 0.30 | 0.32 | 0.23 | 0.48 | 0.22 | 0.16 | * |
