# Supplementary material for: Infestation Pattern and Population Dynamics of the Tropical Bed Bug, Cimex hemipterus (F.) (Hemiptera: Cimicidae) Based on Novel Microsatellites and mtDNA Markers
Source: Insects. 2020 Jul 25;11(8):472. doi: 10.3390/insects11080472 (PMC7469168; doi:10.3390/insects11080472)
Supplement: Supplementary file 1 [file insects-11-00472-s001.zip › insects-875826-supplementary_proof_revised/Supplementary Table S6_rev.docx]

| **Supplementary Table S6: Estimates of evolutionary divergence between haplotypes sequences from concatenated dataset of COI and 16S rRNA genes based on Kimura-2_parameter model.** | | | | | | | | |
| --- | --- | --- | --- | --- | --- | --- | --- | --- |
|  | Hap01 | Hap02 | Hap03 | Hap04 | Hap05 | Hap06 | C. lectularius 1 | C. lectularius 2 |
| Hap01 |  |  |  |  |  |  |  |  |
| Hap02 | 0.002 |  |  |  |  |  |  |  |
| Hap03 | 0.001 | 0.003 |  |  |  |  |  |  |
| Hap04 | 0.001 | 0.003 | 0.002 |  |  |  |  |  |
| Hap05 | 0.001 | 0.003 | 0.002 | 0.002 |  |  |  |  |
| Hap06 | 0.001 | 0.003 | 0.002 | 0.002 | 0.002 |  |  |  |
| Hap07 | 0.001 | 0.003 | 0.002 | 0.002 | 0.002 | 0.002 |  |  |
| C. lectularius 1 | 0.187 | 0.188 | 0.188 | 0.185 | 0.185 | 0.188 | 0.185 |  |
| C. lectularius_2 | 0.188 | 0.190 | 0.190 | 0.187 | 0.187 | 0.190 | 0.187 | 0.003 |
